# A Case of Left-Sided Infective Endocarditis Secondary to Intravenous Drug Use Resulting in Septic Renal Emboli

**DOI:** 10.7759/cureus.71797

**Published:** 2024-10-18

**Authors:** Sachi Patel, Danielle A Rowe, Marutha Arulthasan

**Affiliations:** 1 College of Medicine, American University of Antigua, Osbourn, ATG; 2 Internal Medicine, Richmond University Medical Center, New York, USA

**Keywords:** infective endocarditis, intravenous drug use, mortality, mssa, septic emboli

## Abstract

Methicillin-sensitive *Staphylococcus aureus *(MSSA) is a gram-positive, coagulase-positive coccus implicated in the pathogenesis of infective endocarditis (IE) due to intravenous drug use (IDU). The tricuspid valve is the most commonly affected valve; however, there is an increased incidence of mitral valve involvement. In this case report, we present a 41-year-old female with no known past medical history but a social history significant for IDU who presented with MSSA bacteremia, sepsis, and IE with vegetations on the mitral valve. This patient had no predisposing immunocompromising conditions; however, her repeated IDU history increased her risks for IE. This patient subsequently developed fatal complications of renal septic emboli. She was treated with broad-spectrum intravenous antibiotics, vancomycin, and ceftriaxone, which was de-escalated to nafcillin following blood culture sensitivity data results. She was ultimately transferred to another facility for surgical management of her condition. We write this case report to bring awareness to this rare but fatal condition and to highlight the presentation of mitral valve vegetations.

## Introduction

Infective endocarditis (IE), inflammation of the endocardium, is the most common complication of intravenous drug use (IDU) [1]. It most commonly affects the tricuspid valve due to bacterial access into the venous system. Though rare, the mitral and aortic valves have also been affected [1,2]. While IE can affect immunocompromised patients, it is associated with up to a 100-fold increase in intravenous drug users due to endothelial injury from injected particulate matter, direct injection of contaminated material, and drug-associated vasospasm leading to intimal damage and thrombus formation [3]. Previously, streptococci viridans was the most implicated pathogen in IE, but recently Methicillin-sensitive *Staphylococcus aureus* (MSSA) has been shown to be the leading cause amongst intravenous drug users [1]. Clinically, IE presents with an insidious onset of fevers, malaise, chills, fatigue, and generalized weakness. Thorough history-taking is often the best method to find predisposing conditions and risk factors to aid in diagnosis [4]. Diagnoses include using the modified Duke criteria which include a combination of clinical, laboratory, and imaging findings [4]. A complete blood count often demonstrates leukocytosis; two separate blood cultures are required to be positive for the pathogen; and an echocardiogram will demonstrate evidence of a mass fixed to the valve [4]. Effective treatment following patient stabilization and resuscitation include prolonged antibiotic regimens and cardiothoracic surgical intervention [4]. A common complication in 22-55% of IE cases is systemic embolization; most commonly, the kidneys and spleen are affected; this is due to increased vegetation size and *S. aureus *pathogens [5,6]. Here we present the case of left-sided mitral valve IE in an intravenous drug user caused by MSSA, resulting in renal and splenic septic emboli, requiring surgery.

## Case presentation

This is a case of a 41-year-old female with no known past medical history who presented to the emergency department (ED) by ambulance from home due to worsening weakness and fever. She denied experiencing any headaches, chest pain, shortness of breath, cough, nausea, or vomiting. The patient endorsed a six-pack-year smoking history, social alcohol consumption, and the use of marijuana. She denied intravenous drug use; however, intravenous drug paraphernalia was found in her belongings, and her urine drug screen was positive for cocaine. The patient was normotensive and saturating well at 99% SpO_2_ on room air with a respiratory rate of 18; she was febrile with a temperature of 102.2F. On physical examination, she was alert and oriented only to herself and place, was ill-appearing, and had an unkempt appearance but was in no acute distress. She had cutaneous track marks on her left arm. On cardiovascular examination, a grade III holosystolic murmur over the apex was appreciated. Her pulmonary and abdominal examinations were unremarkable. Cultures were taken as per the hospital's sepsis protocol and broad-spectrum antibiotics with vancomycin and ceftriaxone were initiated for empiric coverage.

**Figure 1:**
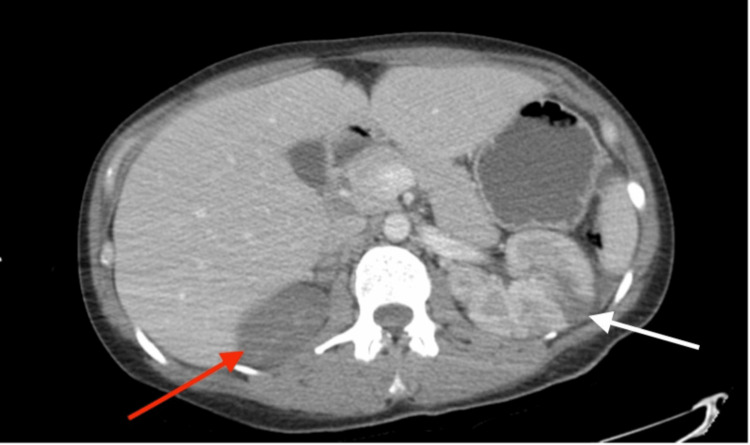
Coronal view of a contrast-enhanced computed tomography (CT) scan of the abdomen and pelvis showing left kidney wedge-shaped hypodensities (red arrow) and non-enhancement of the right kidney (white arrow) suggestive of renal infarction secondary to septic emboli

Initial labs in the ED (Table 1) indicated sepsis with significant leukocytosis WBC of 24.2k/uL and showed elevated inflammatory markers: lactic acid of 3.1 mmol/L, C-reactive protein of 36.9 mg/L, procalcitonin of 37.9 ng/mL, and erythrocyte sedimentation rate of 41 mm/h. The patient was also hyponatremic, with a sodium of 121 mmol/L and elevated renal function tests BUN and creatinine of 31 mg/dL and 1.78 mg/dL, respectively. Hemoglobin was stable at 13.8 g/dL. She was started on 0.9% normal saline at a rate of 100cc/hr. A chest X-ray taken in the ED was unremarkable; however, a computed tomography (CT) of the abdomen/pelvis with contrast (Figure 1) revealed peripheral wedge-shaped hypodensities in the left kidney and spleen, almost complete non-enhancement of the right kidney with questionable lack of flow seen within the distal right renal artery which suggested multifocal infarcts secondary to septic emboli. The patient subsequently had a transthoracic echocardiogram which showed a large mass >10mm on the underside of the mitral valve consistent with vegetation. She was admitted to the critical care unit for further management.

**Table 1:** Laboratory values procured in the emergency department

Investigation	Patients laboratory values	Reference ranges
Hemoglobin	13.8 g/dL	13.5-17.5 g/dL
WBC	24.2 k/uL	4.0-11.2 k/uL
Lactic acid	3.1 mmol/L	0.50-2.20 mmol/L
C-reactive protein	36.9 mg/L	<1.0 mg/L
Procalcitonin	37.9 ng/mL	0.00-0.05 ng/mL
Erythrocyte sedimentation rate	41 mm/h	0-15 mm/h
Serum sodium	121 mmol/L	136-145 mmol/L
Serum blood urea nitrogen	31 mg/dL	7-18 mg/dL
Serum creatinine	1.78 mg/dL	0.7-1.30 mg/dL

Vancomycin was renally dosed due to the patient’s acute renal injury and ceftriaxone was transitioned to meropenem to achieve broader coverage. About 48 hours after admission, blood cultures grew MSSA and antibiotic therapy was narrowed to nafcillin. The patient had four episodes of melena and a four-point drop in her hemoglobin; however, no surgical interventions were required due to a lack of peritoneal signs suggestive of ischemic colitis and any signs of active bleeding at the time of examination by surgery. On hospital day 3, the patient began reporting pain and decreased range of motion in her right upper and left lower extremities, suggestive of polyarthralgia due to septic polyarthritis. On physical examination, she had 2/5 strength in her right upper and left lower extremities and 3/5 strength in the left upper extremity. Four days after admission, she was stable enough for transfer to another hospital for surgery and further management since she was unable to undergo her surgery at the current hospital.

## Discussion

IE is a complex condition involving the cardiac valves, exhibiting a broad array of clinical manifestations and systemic complications. The incidence of IE is on the rise in the United States, with an increasing number of cases attributable to IDU [7]. In the United States from 2000 to 2013, IE hospitalizations rose 38% overall; however, hospitalizations due to IDU-IE increased by 238% and a 12-fold increase was seen between 2007 and 2017 [8]. Most patients present with highly variable clinical manifestations; however, fever, chills, and malaise are the most common non-specific symptoms [9]. IE can progress to exhibit various systemic complications including cardiac complications such as valvular vegetations or abscesses, neurologic complications, systemic immune reactions, and ultimately septic emboli [7]. *S. aureus* is the causative agent in 60-70% of IE caused by IDU and most are methicillin-sensitive organisms [1,5].

While the tricuspid valve is still infected in >50% of IE cases due to IDU, the mitral and aortic valves are implicated in 20-30% [1]. Substances like cocaine and methamphetamines increase systemic afterload resulting in increased turbulence on left-sided heart valves, and our patient’s urine drug screen was positive for cocaine, increasing her risk of mitral valve IE [1]. In a retrospective chart review of 455 patient encounters by Leungsuwan et al. [2] of IE amongst IDU at the University of Kentucky, where overall 9.6% of the study population used cocaine, 29.5% of patients presented left-sided endocarditis and required more impatient valvular surgeries (23.1%) [2]. In the study, the majority of the patients had bacteremia and 25.8% grew MSSA on blood cultures [2]. Additionally, clinical studies have shown that there are worse outcomes and higher mortality with left-sided IE compared to right-sided [1,10]. Left-sided IE overall has poor outcomes due to a higher likelihood of septic shock, renal failure, and perivalvular abscess formation [2,10].

Diagnosis of IE is based on the modified Duke Criteria; major criteria include sustained bacteremia with typical IE organisms and involvement of the endocardium on echocardiogram, while minor criteria include previous heart diseases, fever, vascular, or immunological manifestations. Physical exam findings of Janeway lesions, Osler’s nodes, Roth spots, and splinter hemorrhages are only seen in 5-15% of patients, but more common manifestations include sepsis, embolization, and organ infarcts [1]. Blood cultures, if sent before the administration of antibiotics, have a sensitivity of over 90% and are therefore a great diagnostic tool [1].

IE is complicated by systemic embolization in approximately 22-55% of cases [5]. Renal involvement most often presents as embolic renal infarction, typically caused by Enterococcus or Staphylococcus species, and is associated with larger cardiac vegetations. It should be considered in patients presenting with abdominal symptoms [5,9]. Diagnostic imaging with CT or MRI can help identify the underlying cause of renal involvement. A classic finding of renal infarction is a wedge-shaped perfusion defect on contrast-enhanced CT, evidenced by wedge-shaped hypodensities in the left kidney and complete non-enhancement of the right kidney as in our patient [5].

For most cases, the standard treatment consists of up to six weeks of intravenous targeted antibiotic therapy [7,8]. However, surgical intervention is recommended for certain patients to prevent serious complications. Current guidelines advise surgery in cases of valvular dysfunction, perivalvular extension, infection with a difficult-to-treat pathogen, persistent bacteremia, or fever lasting more than seven days despite appropriate antibiotic therapy [7,8]. Additionally, several studies indicate that vegetation size greater than 10 mm is associated with an increased risk of embolic events and mortality [7,8]. A study by Schran and Barocas supports the favorable outcomes of surgery during the initial hospitalization for left-sided IE [8].

Our patient presented with mitral regurgitation, mitral valve vegetation larger than 10 mm, and septic emboli affecting the kidney and spleen, which prompted surgical intervention along with long-term intravenous nafcillin therapy. Additionally, counseling is a critical component of the treatment plan to ensure antibiotic completion and to reduce the risk of recurrence. In a cohort study from Schranz and Barocas [8] on patients with IE from IDU, it was found that referral for addiction treatment was the only factor, aside from surgery, that significantly improved mortality. Our patient is a young female who falls into the subset of the population at increased risk for IDU-associated IE, and substance abuse is an independent predictor of long-term prognosis [3].

## Conclusions

In conclusion, this case report sheds light on the increasing incidence of IE affecting the mitral valve and its high risk of mortality due to serious complications such as embolization. In conjunction with other reports and cohort studies, this case highlights the need to increase surveillance of left-sided endocarditis as it leads to more impatient valvular surgeries and higher mortality, all while assessing the risks of acquiring it in IDU. The findings in this patient indicate that large left-sided vegetations (>10mm) carry an increased risk for embolization to vasculature in the kidney, spleen, and abdomen and can cause a detrimental stroke. Prompt recognition, appropriate antimicrobial treatment, and surgical intervention are crucial in achieving favorable patient outcomes. Further studies are needed to assess the mechanisms causing this increase as well as focusing on addiction treatment early during hospitalization to improve long-term survival.
